# PP50 Facilitating Academic Life Science Innovation With Early Health Technology Assessment: A Survey Of Potential User Needs And Perceptions

**DOI:** 10.1017/S0266462324002216

**Published:** 2025-01-07

**Authors:** Nick Dragojlovic, Fernanda Nagase, Nicola Kopac, Fan Yi (Grace) Meng, Toluwase Akinsoji, Larry D. Lynd

## Abstract

**Introduction:**

Academic life scientists often struggle to develop and commercialize concrete medical products based on their discoveries. Early health technology assessment (eHTA) can help innovators to define target product profiles (TPPs) with strong value propositions. To understand how eHTA can best help facilitate the clinical translation of university-based inventions, we conducted a survey of stakeholders in the life science innovation ecosystem.

**Methods:**

Our 10-minute online survey includes questions on respondents’ location, organizational affiliations, experiences in health technology development, and awareness and perceptions of eHTA. eHTA is broadly defined as the use of tools from health economics, epidemiology, management, and related disciplines to assess the potential value of a medical product candidate for patients, payers, providers, manufacturers, and other stakeholders. The survey is being advertised using social media and email, and it will be followed up with semistructured interviews. Data on 51 complete responses were summarized using frequency tables and cross-tabulations, and the statistical significance of subgroup differences was evaluated using Fisher’s exact test.

**Results:**

Of 51 respondents, a majority lived in Canada (38/51; 75%) and had an academic affiliation (39/51; 76%). A “lack of commercialization skills among academic life science teams” was identified as a barrier to clinical translation by 41 percent (21/51), though this varied by academic affiliation (33% vs 67%; p=0.051) and industry experience (65% vs 29%; p=0.033). While 31 percent (16/51) reported familiarity with eHTA, this also varied by academic affiliation (23% vs 58%; p=0.033). Only 20 percent (10/51) had previously used eHTA, but a majority expressed an interest in learning more (39/51; 76%) and in using eHTA in the future (31/51; 61%).

**Conclusions:**

Making eHTA more accessible for academic life scientists who lack commercialization experience may mitigate an important barrier to clinical translation of university-developed health technologies. While awareness of eHTA is relatively low in this group, they are interested in learning more about and using eHTA, and efforts should be made to integrate eHTA with existing product development tools like the TPP.

